# Raman and UVN+LWIR LIBS detection system for in-situ surface chemical identification

**DOI:** 10.1016/j.mex.2022.101647

**Published:** 2022-02-26

**Authors:** Clayton S.C. Yang, Dina M. Bower, Feng Jin, Tilak Hewagama, Shahid Aslam, Conor A. Nixon, John Kolasinski, Alan C. Samuels

**Affiliations:** a Brimrose Corporation of America, Sparks-Glencoe, MD, USA; b University of Maryland, College Park, MD, USA; c NASA Goddard Space Flight Center, Greenbelt, MD, USA; d Edgewood Chemical Biological Center, Aberdeen Proving Ground, MD, USA

**Keywords:** Infrared, Spectroscopy, Vibrational, Molecular, plasma

## Abstract

Laser Induced Breakdown Spectroscopy (LIBS) in the Ultra Violet/Visible/Near-IR (UVN) spectral range is a powerful analytical tool that facilitates the interpretation of Raman spectroscopic data by providing additional details in elemental chemistry. To acquire the complete information of molecular vibrations for more accurate and precise chemical bonding and structural analysis, an ideal *in situ* optical sensing facility should be able to rapidly probe the broad vibrational dipole and polarizability responses of molecules by acquiring both Raman scattering and mid-IR emission spectroscopic signatures. Recently, the research team at Brimrose has developed a novel optical technology, Long-Wave IR (LWIR) LIBS. Critical experimental approaches were made to capture the infrared molecular emission signatures from vibrationally excited intact samples excited by laser-induced plasma in a LIBS event. LWIR LIBS is the only fieldable mid-IR emission spectroscopic technique to-date that that offers the same instrumental and analytical advantages of both UVN LIBS and Raman spectroscopy in in-situ stand-off field applications and can perform rapid and comprehensive molecular structure analysis without any sample-preparation.

• A single excitation laser pulse is used to trigger both UVN and LWIR spectrometers simultaneously.
• Time-resolved UVN-LWIR LIBS measurements showed the evolution of both atomic and molecular signature emissions of target compounds in the laser-induced plasma.
• The technique was applied to the characterization of mineral and organic compounds in planetary analog samples.

## Graphical abstract

**Figure:**
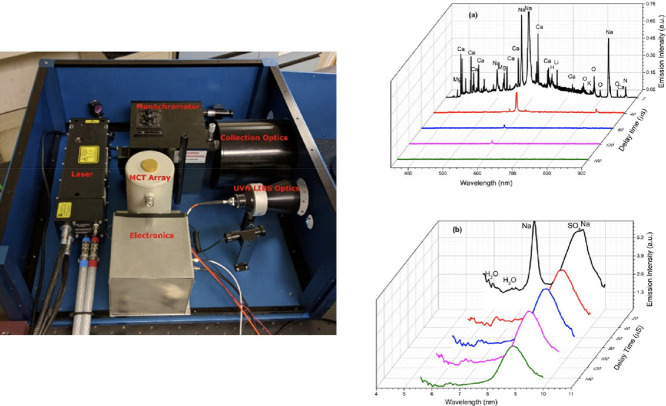

SPECIFICATIONS TABLE

**Table:** 

Subject Area;	Earth and Planetary Sciences
More specific subject area;	*Molecular Spectroscopy*
Method name;	*UVN+LWIR LIBS*
Name and reference of original method;	*1. S. G. Buckley, H. A. Johnson, K. R. Hencken, and D. W. Hahn, Waste* *Management 20, 455 (2000).* *2. K. Loebe, A. Uhl, and H. Lucht, Appl. Opt. 42, 6166 (2003).* *3. L. Nemes, A. M. Keszler, J. O. Hornkohl, and C. G. Parigger, Appl. Opt.* *44, 3661 (2005).* *4. K. Y. Yamamoto, D. A. Cremers, M. J. Ferris, and L. E. Foster, Appl.* *Spectrosc. 50, 222 (1996).* *5. A. V. Pakhomov, W. Nichols, and J. Borysow, Appl. Spectrosc. 50, 880* *(1996).* *6. J. D. Hybl, G. A. Lithgow, and S. G. Buckley, Appl. Spectrosc. 57, 1207* *(2003).* *7. F. C. DeLucia Jr., R. S. Harmon, K. L. McNesby, R. J. Winkel Jr., and A.* *W. Miziolek, Appl. Opt. 42, 6148 (2003).* *8. A. C. Samuels, F. C. DeLucia Jr., K. L. McNesby, and A. W. Miziolek,* *Appl. Opt. 42, 6205 (2003).*
Resource availability;	*N/A*

## Method details

Current advances in laser-based optical spectroscopy demonstrate the effectiveness of non-contact methods for *in situ* and remote sensing of surface deposit composition on planetary bodies. For example, Raman spectroscopy has been chosen for *in situ* planetary NASA missions because it is an information-rich, non-contact, non-destructive method for identifying and characterizing inorganic and organic compounds in a wide variety of planetary materials. Laser Induced Breakdown Spectroscopy (LIBS) in the Ultra Violet/Visible/Near-IR (UVN) spectral range is a powerful analytical tool that facilitates the interpretation of Raman spectroscopic data by providing additional detail in elemental chemistry. Both LIBS and Raman spectroscopy are part of the payloads of NASA's Perseverance rover and on ESA's ExoMars rover [1,2].

However, some challenges remain. For Raman spectroscopy, the spectral signature signals are usually very weak and the sensitivity and accuracy of the surface measurements are strongly influenced by surface shape, roughness, composition irregularity and background emissions (e.g. fluoresce from the surface) [3]. Another challenge for Raman spectroscopy is in distinguishing diagnostic peaks in samples of mixed composition, common for compositionally heterogeneous targets in natural environments. Structural commonalities between compounds can result in similar peak positions of many co-occurring substrates and organic compounds, resulting in the misinterpretation of spectra and possible misidentification [4]. And though UVN LIBS helps to clarify some of ambiguity in the Raman spectra, it is an atomic spectroscopic technology that reveals no information on molecular structures [5].

To acquire the complete information of molecular vibrations for more accurate and precise chemical bonding and structural analysis, it would be ideal for an *in situ* optical sensing facility to rapidly probe the broad vibrational dipole and polarizability responses of molecules by acquiring both Raman scattering and mid-IR emission spectroscopic signatures of inorganic and organic compounds. The main differences between the two major active vibrational spectroscopy techniques: Raman scattering and mid-IR emission spectroscopy is that IR intensities are determined by the change of the molecular dipole moment during a vibrational transition, while the Raman intensities are determined by polarizability changes. Therefore, the intensities and selection rules of vibrational transitions can be very different between Raman and IR. In general, symmetric vibrations and non-polar functional groups of the molecules are better detected in Raman while the asymmetric vibrations and polar functional groups are better detected in IR. Because of these differences, IR and Raman spectra may identify different species in the same sample [6]. However, to apply mid-IR emission spectroscopy as an active *in situ* probe in any field application to-date has been difficult due to the lack of high-speed (nanosecond to microsecond) and efficient excitation and acquisition schemes.

Recently, the research team at Brimrose has developed a novel optical technology, Long-Wave IR (LWIR) LIBS (also known as the Laser-Induced Thermal Emission (LITE)), to capture the infrared molecular emission signatures from vibrationally excited intact samples excited by laser-induced plasma in a LIBS event [5,7]. This innovative mid-IR (3 to 12 µm) emission technology, in which the molecular structure of constituents is revealed by spectrally analyzing the emission profile from a laser-induced plasma on the target surface using mid-IR spectrometry, is the only vibrational-rotational mid-IR emission spectroscopic technique to-date that offers the same instrumental and analytical advantages of both UVN LIBS and Raman spectroscopy for *in situ* field applications [5,[7], [8], [9]]. With all the advantages of the conventional UVN LIBS, the newly developed LWIR LIBS technology is able to provide *in situ*, stand-off (1-6 m), and rapid real-time/near-real-time molecular structural analysis regardless of the physical and optical properties of the sample surface while requiring no need for any sample preparations. The experimental simplicity of LWIR LIBS makes it particularly suitable for various field applications with harsh conditions. For surface reconnaissance applications of solar system bodies, a detection system equipped both Raman and UVN LIBS + LWIR LIBS is a significant prospective tool.

## Simultaneous UVN+LWIR LIBS detection method

**Fig. 1:**
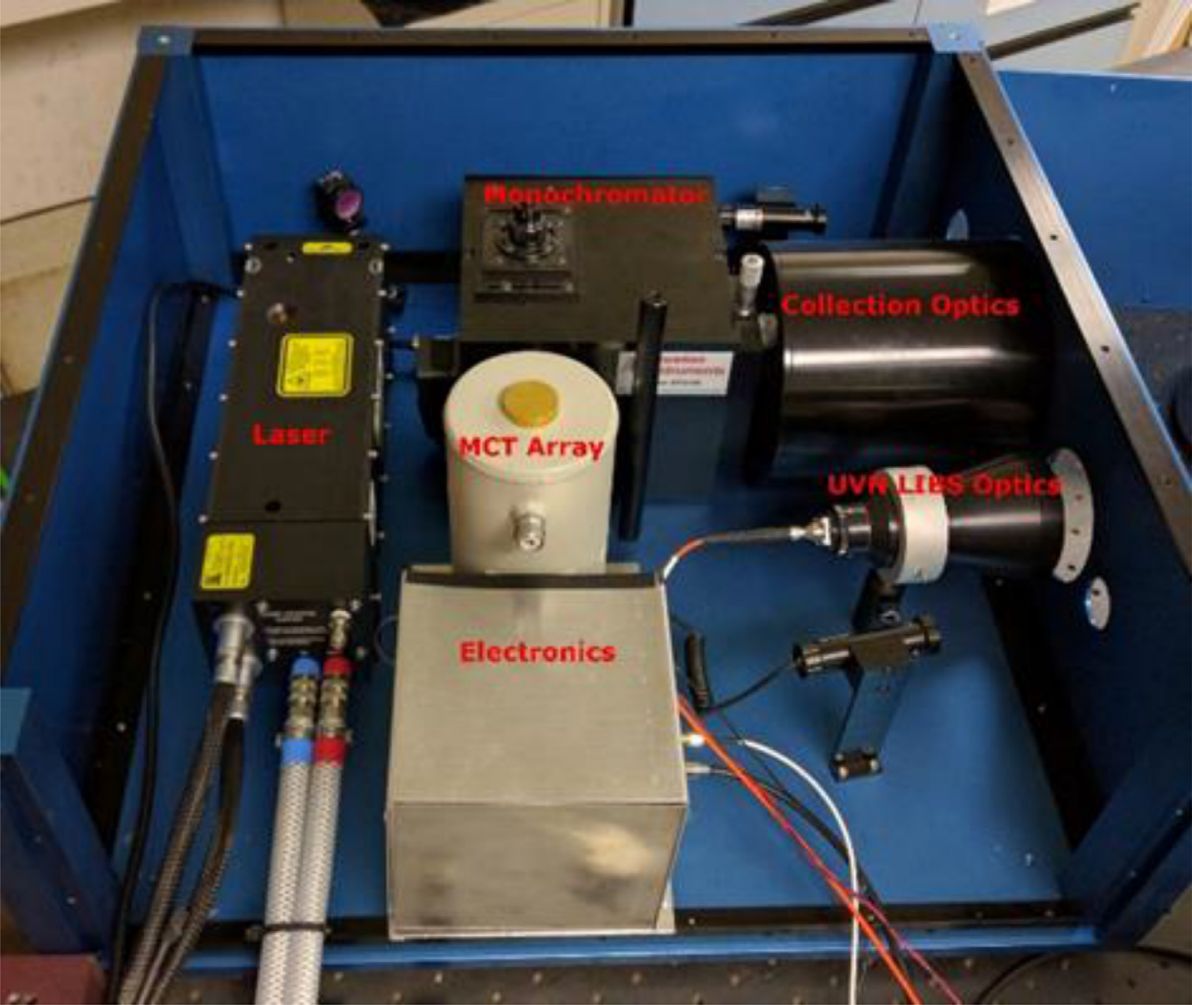
Laboratory setup of the simultaneous UVN+LWIR detection system.

The setup for the simultaneous UVN+LWIR detection system is shown in Fig. 1. The excitation laser for the system is a actively Q-switched Nd:YAG laser CFR400 by Quantel Lasers. It operates at a repetition rate of 10 Hz and pulse energy of 75-110 mJ. A plano-convex lens is used to focus the laser on to the test sample one meter from the instrument. LWIR collection optics is a Cassegrain type telescope with a primary mirror of 6-inch diameter. Emission of the plasma is collected by this telescope and focused onto the input slit of a modified SP2150 Czerny–Turner monochromator by Princeton Instruments with an input focal length of 150mm, grating of 30 grooves/mm, and output focal length of 50mm. The shorter output focal length allows wider spectral range to be acquired and higher optical intensity at the detector pixels. The input slit is set to 0.5 mm wide. The original output focusing lens was replaced with a flat mirror. The output of slit was removed, and a 50 mm focal length lens was used to focus the output onto a liquid-nitrogen-cooled linear LWIR array integrated with ROIC. The linear detection array contains 300 HgCdTe (mercury cadmium telluride, MCT) detectors/pixels with a pitch and height of 50 µm. Analog output of the detection array/ROIC is converted into 16-bit values as each of the pixel output is clocked out by the ROIC.

The visible/NIR emission from the plasma is collected by a pair of 75 mm diameter lenses, one with 1 meter focal length (to collimate collected signal), and a second one with 100 mm focal length) to focus light into a 200 µm core, 0.22 N.A. multimode optical fiber). Output of the optical fiber is fed to a spectrometer (CCS200 by Thorlabs) with a nominal spectral range of 200 to 1000 nm.

**Fig. 2:**
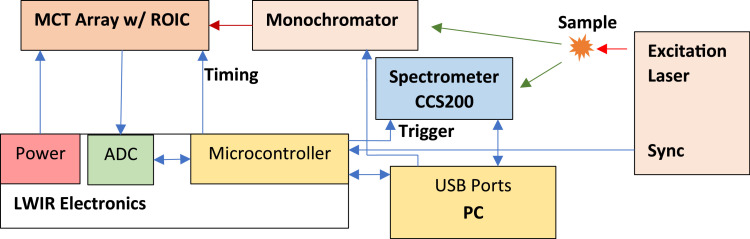
Operation and control diagram of the instrument.

The operation of the instrument and controlled by a computer, as well as a microcontroller (Fig. 2). In preparation for each set of measurements, the PC sends the desired operation parameter to the microcontroller, including LWIR delay time (from excitation laser pulse) and integration time, and Vis/NIR spectrometer delay time. The Vis/NIR integration time is sent to CCS200 directly by the PC via USB port. When the excitation laser emits a laser pulse, an electrical synchronization pulse is also sent to the microcontroller. Upon receiving the pulse, the microcontroller sends trigger pulses to CCS200 and LWIR linear array after their respective delay times, and then stops LWIR linear array integration when integration time is reached. Vis/NIR integration is controlled by the CCS200 spectrometer firmware internally. Upon integration completion, microcontroller clocks out linear array pixel values and digitizes each pixel's output. Vis/NIR spectrometer's digital output is read out by PC via USB port. Typical delay and integration times for LWIR are 20 and 42 µs, respectively, and 3 and 10 µs for Vis/NIR spectrometer respectively.

LWIR linear array output signal contains significant amount of detector dark current and background. Therefore, the background values are measured for the same integration time are measured without the presence of laser pulse before each set of measurement. In addition, LWIR spectrum of a blackbody source of around 600 degree C is also measured to characterize the pixel-to-pixel variation of responsivity, which will be used in post data processing for non-uniformity correction. The combination of background and blackbody measurement and correction constitute a 2-point correction to the acquired LWIR spectrum.

The detection system scans the surface of the solid samples without any sample preparation. Within seconds after the firing of the excitation laser pulse, the detection system acquires both UVN and mid-IR LIBS spectral fingerprints of the targets initiated by the same single laser-pulse-induced micro-plasma on the target surface, analyzes both atomic and molecular chemical composition on these surfaces.

## Validation of the simultaneous UVN + LWIR LIBS detection system on planetary analog samples

Each set of simultaneous UVN + LWIR LIBS emission spectra studied in this work using our lab-based UVN + LWIR LIBS spectrometer was acquired from a single laser pulse-induced plasma on the target surface in ambient air atmosphere unless stated otherwise. All rock and mineral samples were from NASA Goddard Research Center (“SS1 – siliceous sinter from a terrestrial hot spring”, “LTA” and “LT1” – secondary precipitates collected from a terrestrial lava tube) and a GSI 1050-00E mounted 50 specimen collection set (limestone, quartz, gypsum, and basalt) probed “as is” at standoff distance of one meter. The samples were also characterized using a fiber-fed field portable VIS (532 nm) Raman spectrometer (EZ-Raman from TSI, Inc) with a spot size of 50 µm, spectral range between 100 - 4000 cm^−1^, and a resolution of ∼10 cm^−1^.

### SS1 - siliceous sinter

**Fig. 3:**
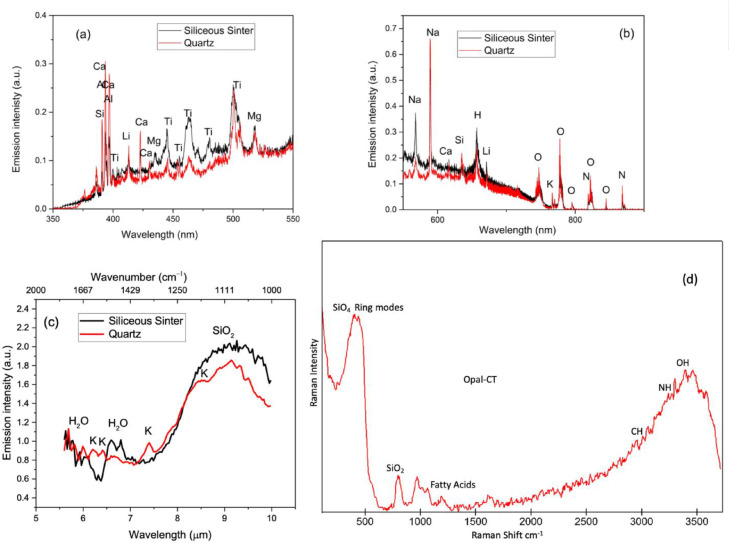
The simultaneous UVN (a)(b) + LWIR (c) LIBS spectra of the siliceous sinter sample SS1 (black line) and quartz (red line) agree with the Raman spectroscopic identification (d) of opal-CT (amorphous silica) composition; also shown are Raman peaks for fatty acids in the sinter sample.

The simultaneous UVN + LWIR LIBS emission spectra of SS1 showed elemental emission signatures in the UVN (Fig. 3a and 3b) such as H (565 nm), Si (390 nm), Ti (445 nm, 455 nm, 464 nm, 480 nm), Na (568 nm, 589 nm), Al (394 nm, 396 nm), N (868 nm), O (777 nm), and Ca [10]. A broad SiO_2_ vibrational emission feature (Si-O-Si asymmetric stretching) around 9.2 µm (1086 cm^−1^) [8] and a strong emission features around 6.6 µm (1515 cm^−1^) (Fig. 3c) can be clearly identified in the mid-IR spectrum. In the UVN LWIR LIBS emission spectra of quartz (Fig. 3), a SiO_2_ crystal, the Ca, Al, Si atomic features in the UVN and the broad SiO_2_ (Si-O-Si stretching) molecular vibrational feature (9.2 µm, 1086 cm^−1^) in the mid-IR are clearly visible along with four K atomic signature emissions (6.23 µm, 6.44 µm, 7.4 µm, 8.54 µm) in the infrared resulting from the high-lying Rydberg transitions.

The major differences between the spectra of SS1 and quartz are the strong atomic emissions features of Ti at 445 nm, 455 nm, 464 nm and 480 nm and the strong mid-IR emission band around 6.6 µm (1515 cm^−1^). The 6.6 µm (1515 cm^−1^) band is the P branch of the molecular bending rotational-vibrational feature of water indicating the presence of bound water in the mineral sample [5]. The band center of this molecular bending rotational-vibrational band of water at 6.3 µm (1587 cm^−1^) is also clearly observed in the LWIR LIBS spectrum of the “siliceous sinter” sample. This water band centered at 6.3 µm (1587 cm^−1^) is due to the scissoring bending of the two OH bonds and is unique to water and is not due to other hydroxyl groups. The presence of this band as shown in the mid-IR LIBS spectrum of the minerals samples is a strong evidence for the presence of water molecules, and so it is not due to any other hydroxyl groups, in the opaline silica (SiO_2_ + nH_2_O). This is in agreement with the Raman measurements (Fig. 3) in which the spectra for SS1 show the broad features of opaline silica between 200 cm^−1^ and 490 cm^−1^ and OH between 3000 cm^−1^ and 3600 cm^−1^ [11]. In addition, the UVN + LWIR LIBS spectral signatures also indicate that SS1 contains abundant Ti impurities, which was not observed in the Raman spectra. The accumulation of Ti observed in the opaline silica is very common and often interpreted as evidence for the dissolution of basaltic materials by acidic fluids that concentrate the relatively insoluble SiO_2_, TiO_2_ or TiCl_4_ components [12]. The Raman measurements also detected signatures for fatty acids between 900 cm^−1^ and 1750 cm^−1^, 2957 cm^−1^ (CH_3_), and 3297 cm^−1^ (N-H stretch) attributed to the microbial communities known to colonize the sinter deposits [13,14].

### Sulfate precipitates – LTA and LT1

**Fig. 4:**
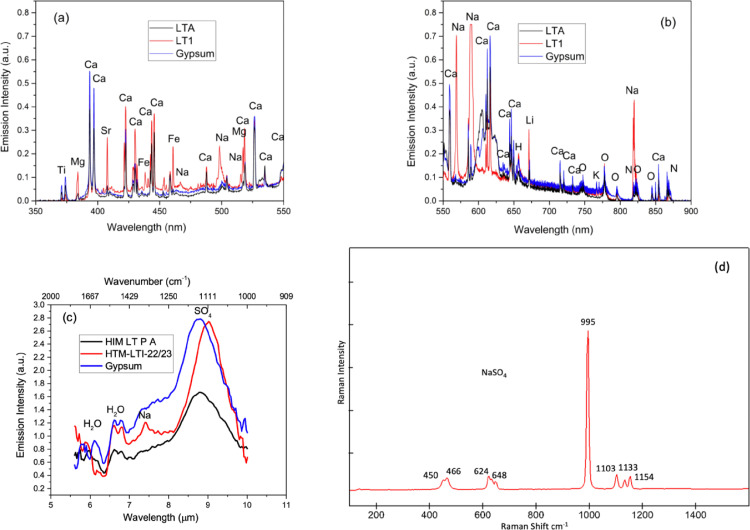
The simultaneous UVN (a)(b) + LWIR (c) LIBS spectra of sulfate-rich samples LTA (black line), LT1 (red line) and Gypsum standard (blue line) agree with the Raman spectroscopic identification of Na-sulfate (thenardite) in the samples.

In contrast, the UVN LIBS emission spectra of the LT1 sample showed strong Na, Mg, and Sr (407 nm) atomic signature emissions instead of the Ca observed in the spectrum of the gypsum standard and LTA samples. The LWIR LIBS emission spectrum of the LT1 shows strong vapor phase bending features of bound water molecules centered at 6.3 µm (1587 cm^−1^) and a strong Na atomic signature emission at 7.43 µm resulting from the high-lying Rydberg transitions of 8^2^F_7/2_ →6^2^D_5/2_. The mid-IR LIBS emission spectrum of LT1 also showed an intense vibrational feature of SO_4_ asymmetric stretching at 8.9 µm (1123 cm^−1^) which was a little red-shifted in comparison with that of CaSO_4_ observed in the gypsum and LTA samples. Similar red-shifting of the SO_4_ asymmetric stretching band from that of CaSO_4_ was also observed in the FTIR study of Na_2_SO_4_ and CaSO_4_ [15,16]. The Raman spectroscopy measurements of LT1 identified thenardite, a typically hydrated Na_2_SO_4_ with diagnostic peak shifts 450 cm^−1^, 466 cm^−1^, 624 cm^−1^, 648 cm^−1^, 995 cm^−1^, 1103 cm^−1^, 1133 cm^−1^, and 1154 cm^−1^ (Fig. 4). The UVN + LWIR LIBS spectral signatures of LT1 agree with the Raman identification of the NaSO_4_, and the lack of OH signatures in the Raman spectra could be attributed to loss of water on the mineral's surface. Hydrated sulfates in the natural environment are metastable and can also change hydration state depending on environmental conditions [17]. The LIBS data also provided a higher level of compositional detail by detecting other cations Mg, Ca, and Sr that are present in low concentrations but are not readily observable as part of the mineral structure in the Raman spectra [18].

### Time-resolved UVN + mid-IR LIBS spectrum of sample LT1

**Fig. 5:**
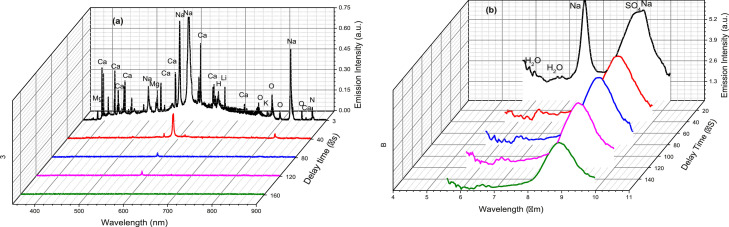
The simultaneous UVN (a)(b) + LWIR (c) LIBS spectra of LT1 at 5 different delay times of 5 µs, 40 µs, 80 µs, 120 µs, 160 µs.

Another advantage of the UVN + LWIR LIBS technique is its ability to analyze the evolution of both atomic and molecular signature emissions of the target compounds in the laser-induced plasma. The UVN + LWIR LIBS emission spectra of LT1 at different delay times are shown in Fig. 5. Within the 355-900 nm region, the prominent emission features from Ca (393nm, 397 nm), and Na (568 nm, 589 nm, 847 nm), N (868 nm), and O (777 nm) are clearly identifiable at 3 µs. The intensities of those atomic emission lines decreased with time and generally became insignificant after 40 μs, which is consistent with the atomic line emission lifetime timescale (typically 0.1 to tens of microseconds) observed in UVN LIBS studies [5]. In the mid-IR region, the Na atomic emissions at 7.43 µm and 9.17 µm (similar to those visible counterparts) also vanished after 40 μs while the water vapor molecular vibrational-rotational emission bands centered at 6.3 µm (1587 cm^−1^) and the vibrational features of SO_4_ asymmetric stretching at 8.9 µm (1123 cm^−1^) decayed at a much slower rate, and all the molecular emission features were still distinguishable even beyond 200 μs. Similar slow decay rates were also observed from other molecular mid-IR LIBS vibrational emission features of solid samples, e.g. NH_4_ asymmetric stretching at 7.5 μm and ClO_4_ asymmetric stretching and deformation bands around 9 μm of ammonium perchlorate NH_4_ClO_4_ samples [8], and water HOH bending in vapor phase around 6.3 μm of ice samples [5]. The time-resolved spectra of mid-IR emissions showed that complex molecular species in the laser-induced plasma plume lingered for a few hundred microseconds after the initiation of the plasma, while their atomic counterparts last only tens of microseconds. This difference in emission time scale can be exploited to improve the sensitivity and specificity of chemical elemental and molecular functional group identification.

## Conclusion

Distinct atomic and molecular emission signatures of the target compounds measured simultaneously in UVN (200 to 1000 nm) and LWIR (5.6 to 10 µm) spectral regions are readily detected and identified. Major molecular constituents of the samples, such as CO_3_ (carbonates), SiO_2_, SO_4_ (sulfates) and H_2_O, were straightforwardly identified without the need of employing complex data processing. Our studies illustrate that the combination of atomic emission signatures derived from conventional UVN LIBS and mid-IR fingerprints of intact molecular entities determined from LWIR LIBS adds robustness in identification algorithms to the current available laser ablation spectroscopy techniques for in situ chemical identification and detection. LWIR LIBS is the only vibrational-rotational mid-IR emission spectroscopic technique to-date that offers the same instrumental and analytical advantages of both UVN LIBS and Raman spectroscopy in on-site stand-off field applications.

## Declaration of interests

The authors declare that they have no known competing financial interests or personal relationships that could have appeared to influence the work reported in this paper.
